# Research on Quantitative Characterization Model of Compressive Strength or Elastic Modulus of Recycled Concrete Based on Pore Grading

**DOI:** 10.3390/ma18010003

**Published:** 2024-12-24

**Authors:** Fuwei Xu, Hongke Pan

**Affiliations:** 1 School of Architecture and Engineering, Hubei University of Arts and Sciences, Xiangyang 441053, China; 10916@hbuas.edu.cn; 2 College of Architecture and Engineering, Xinyu University, Xinyu 338004, China

**Keywords:** pore grading, recycled concrete, compressive strength, elastic modulus, quantitative characterization model

## Abstract

The influence of different pore sizes on the compressive strength and elastic modulus of recycled concrete is an important issue in the academic circle. Aiming at this problem, a quantitative characterization model of the compressive strength and elastic modulus of recycled concrete based on pore grading was established in this paper. The compressive strength, elastic modulus, porosity and distribution of pore size of recycled concrete were measured by a concrete test and nuclear magnetic resonance technology, and the influences of different pore sizes on the compressive strength and elastic modulus of recycled concrete were analyzed, and the rationality of the quantitative characterization model was verified. The results showed that the compressive strength and elastic modulus of recycled concrete decreased with the increase in the recycled coarse aggregate replacement rate, and the decrease was more obvious with the increase in the substitution rate. The peak pore diameter in the distribution curve of porosity and pore diameter of recycled concrete increased, and the proportion of pore diameter above 50~200 nm and 200 nm also increased. The pore sizes, including those below 20 nm and 20~50 nm, had a positive correlation with the compressive strength and elastic modulus of recycled concrete, while the pore sizes, including those above 50~200 nm and 200 nm, had a negative correlation with the compressive strength and elastic modulus of recycled concrete. The test results of recycled concrete verify that the quantitative characterization model could better characterize the compressive strength and elastic modulus of recycled concrete.

## 1. Introduction

Compared with ordinary concrete, due to the addition of recycled coarse aggregate, the content of new and old cement mortar and interfacial transition zone of recycled concrete increase, and the content of various pores and micro-cracks caused by stress and non-stress factors are more, and their distribution is more complex, which also had a greater impact on the macro-mechanical properties of recycled concrete [1]. In 1896, Feret [2] first put forward the concrete strength formula ($\sigma =K\cdot (\frac{c}{c+w+a}{)}^{2}$) and carried out a quantitative analysis of pores and concrete strength. Since Professor F.H. Wittmann [3] proposed the concept of “poresology” in 1980, scholars at home and abroad have carried out relevant studies on the pore size distribution (grading) and pore morphology of concrete pores and their effects on the macroscopic mechanical properties of concrete. When analyzing the recent development direction of concrete science and technology, Wu Zhongwei [4] pointed out that pores with different pore sizes had different degrees of influence on the strength of concrete. According to the harm of different pore sizes to the strength of concrete, the pores were divided into four types: harmless pores, less harmful pores, harmful pores and multiple harmful pores. Reducing the content of harmful pores and multiple harmful pores was conducive to improving the strength of concrete. Wittmann [3] proposed a relationship between pore structure and tensile strength on the basis of relevant research theories of material science and fracture mechanics. Atize [5] believed that it was more accurate to replace the initial maximum defect size with the square root of the mean pore size, and improved Wittmann’s formula. In order to deeply analyze the relationship between pore grading and macroscopic mechanical properties of concrete, scholars from different countries started from the microstructure model of cement-based composite materials, and on the basis of different assumptions, explored and proposed different relationship models from different sides to explore the relationship between concrete strength and pore grading [6]; for example, in the classification and classification model proposed by Powers–Brunauer, Feldman–Sereda, Munchen, Kondo Liichi–Omon Masaji, Boot, etc., the pores were divided into different types according to the pore size, and the influence of different pore sizes on the macroscopic mechanical properties of concrete was studied. Compared with ordinary concrete, the pore distribution of recycled concrete was complicated, the pore shape was different, the pore size span was larger, and the macroscopic mechanical properties of recycled concrete were inevitably related to the pore grading. The results of the experimental study on the pore structure of recycled aggregate concrete by Zhang Jianbo [7] found that the increase in the water–cement ratio, recycled aggregate content and the water-reducing agent would increase the porosity of recycled concrete. The experimental analysis results of different pore sizes and strengths of recycled coarse aggregate concrete by Zhang Jinxi et al. [8] showed that the pore content of concrete with pore sizes greater than 10 μm and smaller than 10 μm tends to increase with the increase in recycled coarse aggregate content. Both the increase in porosity and the increase in pore sizes greater than 10 μm will reduce the compressive strength of concrete. Xue Cuizhen [9] made construction waste composite powder materials by crushing and grinding waste clay bricks and analyzed the influence of the addition of construction waste composite powder materials on the compressive strength and pore parameters of concrete. The prediction model of concrete compressive strength and pore parameters (total pore area, total pore volume, average pore size, porosity) and number of pore size distribution (gel pores, transition pores, capillary pores, large pores) were established. The results showed that the correlation coefficient between average pore size and pore content and compressive strength is the highest. The regression relationship between mean pore size (*D_m_*) capillary content (*V*) and compressive strength (*σ_c_*), as shown in Equation (1), was proposed.

(1) $$f_{c}=19.167+12.42V+0.269V^{2}-1.565D_{m}+0.096D_{m}^{2}+0.587VD_{m}$$

Yin Zhigang [10] proposed the quadratic relation of mean pore size and compressive strength, as shown in Equation (2), to study the effect of pore structure on freeze-thaw durability of recycled aggregate pervious concrete.

(2) $$f_{c}=159.81-39.31D_{m}+2.75D_{m}^{2}$$

Chen Haiyu et al. [11] studied the influence of porosity and pore parameters (most available pore size, mean pore size, critical pore size) on the compressive strength and elastic modulus of recycled concrete and proposed a quadratic quantization characterization function on the interaction between porosity and pore parameters on the compressive strength and elastic modulus of recycled concrete, which was used to predict the compressive strength and elastic modulus of recycled concrete.

Zhang Yudong et al. [12] analyzed the pore structure of recycled concrete and found that the smaller the volume of bubbles, the larger the proportion of beneficial bubbles and the more beneficial to concrete.

In the study of concrete, grading mainly refers to the classification of coarse aggregate, fine aggregate, cement and other major components according to the particle size, which determines the particle content at all levels and adjusts the particle content at all levels according to the requirements. The pore grading of concrete was mainly the combination of pore content of different pore sizes in recycled concrete.

The existing models of the relationship between pore parameters and concrete strength were mainly established on the basis of total porosity or porosity of a certain type of pore size. Like concrete, the compressive strength or elastic modulus of recycled concrete was obviously not only affected by a certain type of pore size or a certain pore parameter but was inevitably related to the pore grading of different pore sizes. Therefore, it was necessary to analyze the influence of different pore sizes on the macroscopic mechanics of recycled concrete and establish a quantitative characterization model of macroscopic mechanical properties of recycled concrete with hole grading, which was helpful to further clarify the correlation between different pore sizes and compressive strength or elastic modulus of recycled concrete from the macro and micro levels and provide references for the promotion and application of recycled concrete.

## 2. Establishing Quantitative Characterization Model of Compressive Strength or Elastic Modulus

The influence of pores with different pore sizes on the macroscopic mechanical properties of recycled concrete was definitely different. The existing pore-strength relationship models are all quadratic function relations, so the relationship between pores and strength should conform to the quadratic function relationship. Therefore, the pores of recycled concrete were divided into the pore grading of different pore sizes according to the classification method proposed by Professor Wu Zhongwei. Considering the influence of different pore sizes on the macroscopic mechanical properties of recycled concrete, a quantitative characterization model of macroscopic mechanical properties of recycled concrete based on pore grading was established, as shown in Equation (3).

(3) $$\left\{\begin{array}{l} f_{c}=A_{0}+A_{1}P_{<20}+A_{2}P_{<20}^{2}+A_{3}P_{20–50}+A_{4}P_{20–50}^{2}+A_{5}P_{50–200}+A_{6}P_{50–200}^{2}+A_{7}P_{\geq 200}+A_{8}P_{\geq 200}^{2}\\ E=B_{0}+B_{1}P_{<20}+B_{2}P_{<20}^{2}+A_{3}P_{20–50}+A_{4}P_{20–50}^{2}+A_{5}P_{50–200}+A_{6}P_{50–200}^{2}+A_{7}P_{\geq 200}+A_{8}P_{\geq 200}^{2} \end{array}\right.$$

where *f_c_* is the compressive strength of recycled concrete, *E* is the elastic modulus of recycled concrete, *P* is the porosity of different pore sizes, and *A* and *B* are the undetermined coefficients.

## 3. Test Study of Recycled Concrete

### 3.1. Test Material

(1) Coarse aggregate

**Figure 1:**
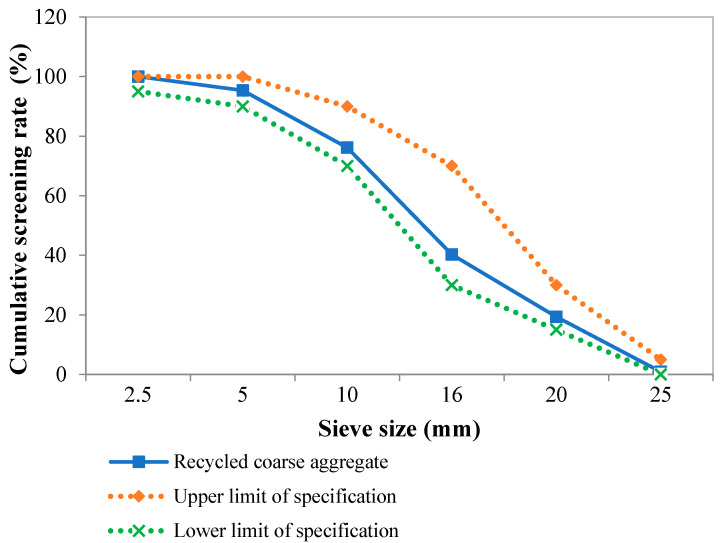
Grading curve of crushed aggregate.

Recycled coarse aggregate (RCA) was obtained from waste concrete during the reconstruction of Changhong Road in Xiangyang City. Waste concrete was broken with a Liyuan HPEF-125 × 100 sealed environment-friendly jaw crusher (manufactured by Liyuan Mining and Metallurgy Equipment Co., Ltd., Nanchang City, China) in the laboratory, whose discharge particle size ranges from 0 to 25 mm. The aggregate grading curve after crushing is within the upper and lower limits specified in the People’s Republic of China standard “Pebbles and crushed stone for construction (GB/T 14865-2022)” [13], as shown in Figure 1, and the basic physical properties of the recycled coarse aggregate and natural coarse aggregate (NCA) are shown in Table 1. Aggregate with a particle size of more than 5 mm was retained as RCA, which was mixed with NCA (the NCA was the standard aggregate used in the laboratory, which met the People’s Republic of China standard “Pebbles and crushed stone for construction (GB/T 14865-2022)”) in a certain proportion to form the aggregate of recycled concrete, and the grading of aggregate was continuous.

(2) Fine aggregate

The fine aggregate was the standard sand special for the laboratory. According to the inspection method stipulated in the People’s Republic of China national standard “Standard for the Quality and Inspection Methods of Sand and Stone for Ordinary Concrete (JGJ52-2016)” [14], the apparent density of the standard sand was 2430 kg/m^3^, the fineness modulus (*M*_x_) of sand calculated to the percentage of sieve residue was 2.58, the mud lump content was 0.18% and the water absorption rate is 1.9%. It was graded in Zone 2 and graded well.

(3) Test cement

The experimental cement was P.O42.5 ordinary Portland cement produced by Huaxin Cement Co., Ltd., Wuhan, China. Its basic physical properties and main chemical mineral composition are shown in Table 2 and Table 3, which meet the standard requirements of the People’s Republic of China’s national standard “ordinary Portland cement (GB/175-2020)” [15]. Its stability also meets the requirements.

(4) Test water

The test water for the production and maintenance of recycled concrete was Xiangyang residents’ drinking water, which meets the requirements of the People’s Republic of China’s national standard “Concrete Mixing Water Standard (JGJ63-2006)” [16].

The test superplasticizer was a Liba brand high-efficiency superplasticizer, its water-reducing efficiency was 18–25%, its fineness was 0.315 mm (less than 10%) and its content was 0.1~0.3%. The performance indicators of the superplasticizer meet the requirements of the People’s Republic of China’s national standard “concrete admixture” (GB8076-2008) [17].

### 3.2. Mix of Recycled Concrete

According to the People’s Republic of China standard “Common concrete mix design Code (JGJ55-2019)” [18], the strength label of recycled concrete in this test was C30, the slump design range was 55~70 mm, the water–cement ratio of recycled concrete was 0.4 and the replacement ratio of RCA was 10%, 20%, 30%, 40% and 60%. Test blocks of recycled concrete were marked according to the numbered forms of RC10, RC20, RC30, RC40 and RC60. respectively. The mix of recycled concrete is shown in Table 4.

### 3.3. Production of Recycled Concrete Test Blocks

The recycled concrete test blocks were produced according to the test method of the People’s Republic of China standard “Standard for test method of performance on ordinary fresh concrete (GB/T 50080-2016)” [19]. The recycled concrete mixture was mixed with a wheel-type vertical concrete mixer TJ2000 (manufactured by Zhengzhou Hongfu Machinery Equipment Co., Ltd. in Zhengzhou City, Henan Province, China), and then put into a mold with a size of 100 mm × 100 mm × 100 mm. On the ZP-0.8 × 0.8 vibration platform (manufactured by Xinxiang Hongda Vibrating Equipment Co., Ltd. in Xinxiang City, Henan Province, China), 3 groups of recycled concrete with each number were made, and each group had 6 test blocks. The reclaimed concrete test blocks after mold disassembly were placed in the YH-40B cement standard curing box (manufactured by Qinxing Technology Development Co., Ltd., Ankang City, Shanxi Province, China) in a constant temperature and humidity environment of 20 ± 2 °C and not less than 95% humidity for 28 days.

### 3.4. Test Machine and Scheme of Recycled Concrete Compressive Strength and Elastic Modulus

#### 3.4.1. Test Machine

The testing machine of recycled concrete compressive strength was the YAW-2000D automatic control hydraulic servo pressure testing machine (manufactured by Jinan Chenxin Testing Machine Manufacturing Co., Ltd., Jinan City, China). Its maximum measurement pressure was 2000 kN, and the test force error range was −1% ~1%. The pressure measurement range was 1% to 100% F.S, the resolution of the test force was 0.01 kN and the displacement measurement resolution was 0.01 mm. The loading rate range was from 600 N/s to 60 kN/s, which was automatically controlled and displayed by computer.

The testing machine of recycled concrete elasticity modulus was the DT-12 dynamic compliance meter (manufactured by Tianjin Huida Experimental Instrument Factory, Tianjin City, China). Its main technical indicators were as follows: the measurement frequency range was 100~10 kHz, the measurement error was less than 2%, the frequency sensitivity was 1 Hz and the output power is 0~15 W. Its output mode was as follows: digital tube display, oscilloscope tube graphic display and printer printing. Its working conditions were as follows: working temperature 0~40 °C and relative humidity below 90%. Its working power supply was an AC220 V ± 10%, 50 Hz, 60 W below, and the power can work continuously for 8 h.

#### 3.4.2. The Test Scheme of Compressive Strength and Elastic Modulus

After the recycled concrete test block had reached the curing age, the compressive strength and elastic modulus were measured according to the test methods specified in the standard requirements of the People’s Republic of China national standard “Standard for test methods of concrete physical and mechanical properties (GB/T 50081-2019)” [20]. During the measurement of the recycled concrete compressive strength, the upper and lower surfaces of the test block were not coated with lubricant and were directly in contact with the upper and lower steel plates. The loading speed was 5 kN/s (corresponding to 0.5 MPa), the loading speed was constant and continuous, the concrete specimen was loaded to the loss of bearing capacity and the maximum compressive strength was measured. The test data of recycled concrete compressive strength were converted into the compressive strength according to Equation (4) [7].

(4) $$f_{c}=0.95\times \frac{F_{N}}{A}$$

where *F_N_* is the axial pressure and *A* is the cross-sectional area of the test block

During the measurement of the recycled concrete elastic modulus, after the installation and debugging of the measurement instrument was completed, the recycled concrete test block was filled according to the requirements, the power was switched on and the test was entered into the manual/automatic test state. When the digital display of the instrument showed “00000000”, the measurement of the recycled concrete elastic modulus was carried out by pressing the single key, and the test results were output each time.

### 3.5. Testing Machine and Scheme of Recycled Concrete Pore Grading

#### 3.5.1. Test Instrument

**Figure 2:**
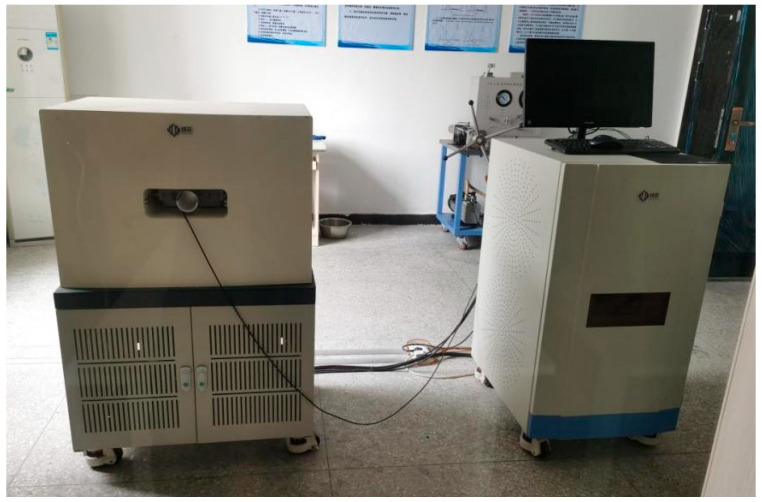
Newmai MesoMR-60S nuclear magnetic resonance spectrometer.

The test instrument of recycled concrete pore grading was the Shanghai Newmai MesoMR-60S nuclear magnetic resonance spectrometer (manufactured by Shanghai Newmai Electronic Technology Co., Ltd., Shanghai City, China), as shown in Figure 2. The main parameters of the spectrometer’s CPMG sequence are shown in Table 5. The pore type was assumed to be cylindrical, and the Coates model was adopted in the permeation model.

#### 3.5.2. The Test Scheme of Pore Structure

**Figure 3:**
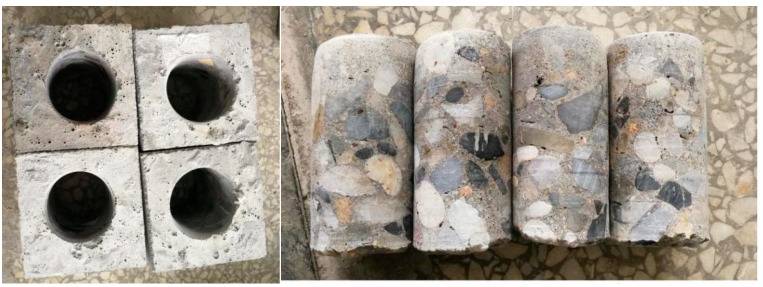
The sample cores of recycled concrete.

In order to accurately determine the pore size distribution of the recycled concrete, the test scheme of recycled concrete hole grading was as follows: Firstly, portable core drilling and sampling equipment was used to drill a cylindrical core with a diameter of 50 mm from the recycled concrete test block. There were four sample cores of recycled concrete with each recycled coarse aggregate replacement rate, and the obtained sample cores are shown in Figure 3. Secondly, the recycled concrete core was immersed in water until the core was saturated with water. Thirdly, the water on the surface of the sample core was dried with a wet towel before the hole measurement, and the pore diameter distribution of the recycled concrete sample core was tested with the nuclear magnetic resonance instrument. The pore diameter distribution of the recycled concrete with each regenerated coarse aggregate replacement rate was taken as the average value of the test results of the four sample cores.

## 4. Results and Discussion

### 4.1. Test Results and Discussion of Compressive Strength or Elastic Modulus

**Figure 4:**
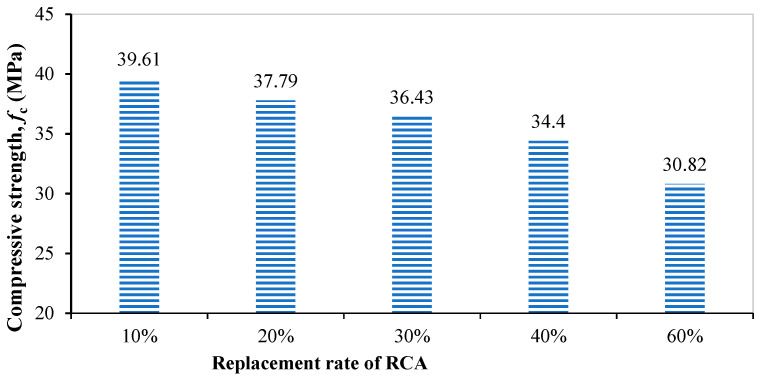
Compressive strength of recycled concrete with different replacement rate of recycled coarse aggregate.

**Figure 5:**
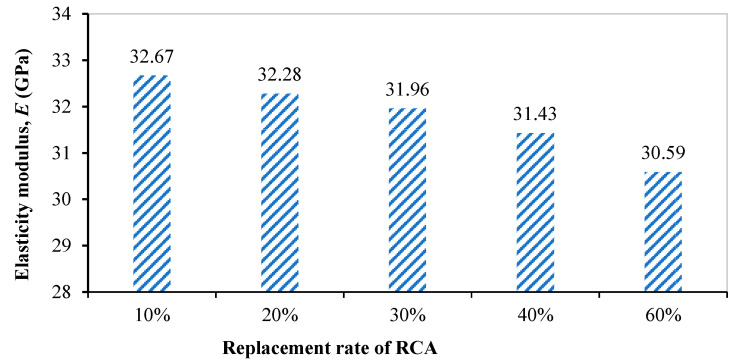
Elasticity modulus of recycled concrete with different replacement rate of recycled coarse aggregate.

After 28 days of curing in the curing box, the recycled concrete test block was respectively tested for compressive strength and elastic modulus, according to the test scheme. Two groups of six test block samples were made of recycled concrete with each replacement rate. The compressive strength and elastic modulus of all six test blocks were tested, and the average values and standard deviations of the compressive strength and elastic modulus were obtained, as shown in Table 6. The final test result was the average value of the test values. The test results of the compressive strength and elastic modulus are shown in Figure 4 and Figure 5, respectively. The test results showed that the compressive strength and elastic modulus of recycled concrete decrease with the increase in the recycled coarse aggregate replacement rate. According to the calculation from Equation (4), the compressive strength of ordinary concrete labeled C30 was 38 MPa. Compared with ordinary concrete, when the replacement rate of recycled coarse aggregate was low, the strength decrease was not obvious and some even had a slight increase, but with the increase in the replacement rate of recycled coarse aggregate, the compressive strength and elastic modulus decreased significantly.

### 4.2. Test Results and Discussion of Pore Grading

**Figure 6:**
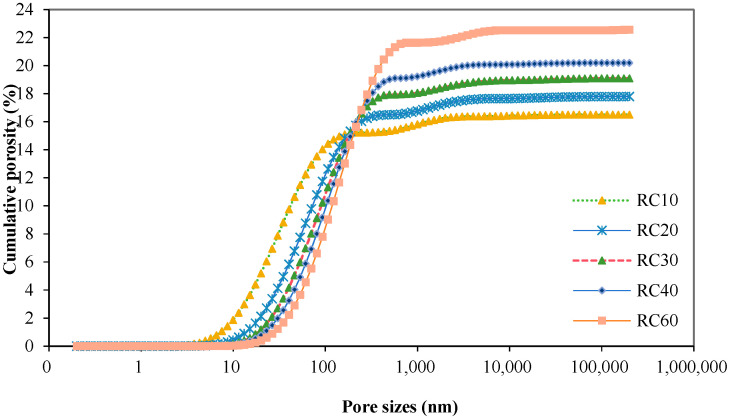
Cumulative porosity curve of recycled concrete.

**Figure 7:**
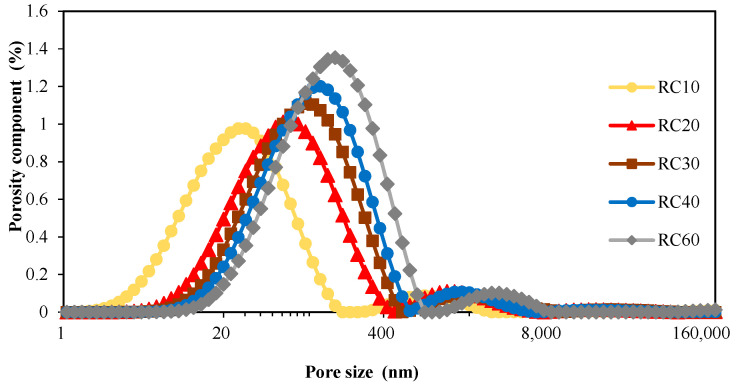
Pore size distribution curve of recycled concrete.

After the recycled concrete sample core was immersed in water to the state of water saturation, the pore grading of the recycled concrete sample core was determined in the nuclear magnetic resonance meter according to the pore measurement scheme. The final test result was the average value of the measured sample core, and the measured aperture distribution results are shown in Figure 6 and Figure 7. The test results showed that the porosity of recycled concrete and the peak pore size in the pore size distribution increased with the increase in the recycled coarse aggregate substitution rate.

**Figure 8:**
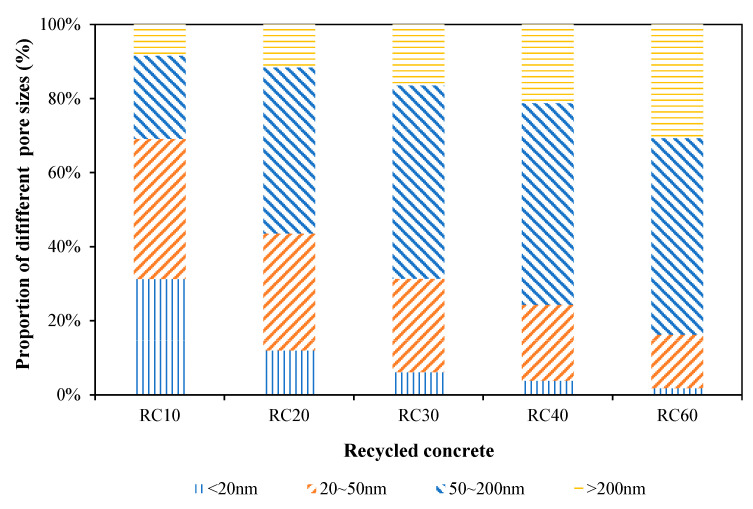
Proportion of different pore sizes.

According to the classification method of pores proposed by Professor Wu Zhongwei [4], the pores were divided into four categories: harmless pore (<20 nm), less harmful pore (20~50 nm), harmful pore (50~200 nm) and multi-harmful pore (≥200 nm). The classification results are shown in Table 7. The percentage of various types of pores is shown in Figure 8. The classification results showed that with the increase in the recycled coarse aggregate substitution rate, the distribution law and proportion of harmful pores and multiple harmful pores in the recycled concrete were increasing, which also led to the continuous reduction in compressive strength and elastic modulus of the recycled concrete.

## 5. Influence Analysis Between Different Pore Sizes and Compressive Strength or Elastic Modulus of Recycled Concrete

### 5.1. Correlation Analysis

**Figure 9:**
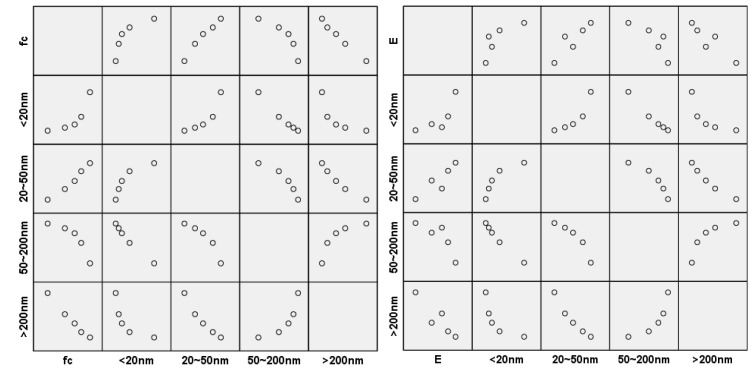
Scatter diagram of different pore sizes between compressive strength and elastic modulus.

Correlation analysis was conducted on the data of compressive strength or elastic modulus of recycled concrete with different pore sizes in Table 8. The correlation is shown in Table 6, and the resulting scatter diagram is shown in Figure 9. The scatter diagram results showed that there was a nonlinear relationship between different pore sizes and the compressive strength or elastic modulus of recycled concrete. The correlation between the pore size below 20 nm or 20~50 nm and compressive strength or elastic modulus was a nonlinear positive correlation, while the correlation between the pore size of 50~200 nm or above 200 nm and compressive strength or elastic modulus was a nonlinear negative correlation. The correlation data in Table 8 also showed that the pore size below 20 nm or 20–50 nm was positively correlated with compressive strength and elastic modulus, the pore size of 20~50 nm was more correlated with compressive strength or elastic modulus and the pore size between 50 and 200 nm or above 200 nm was negatively correlated with the compressive strength or elastic modulus. The pore size above 200 nm was more correlated with compressive strength or elastic modulus.

### 5.2. Influence Analysis Between Pore Size and Compressive Strength or Elastic Modulus

**Figure 10:**
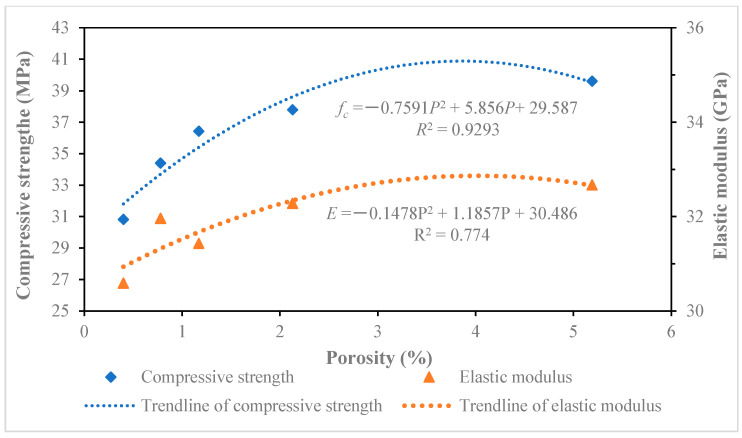
Effect of pores below 20 nm on compressive strength and elastic modulus of recycled concrete.

**Figure 11:**
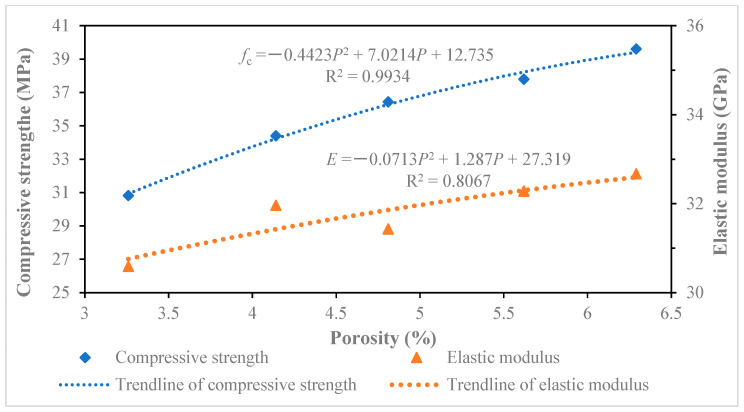
Effect of pores 20~50 nm on compressive strength and elastic modulus of recycled concrete.

**Figure 12:**
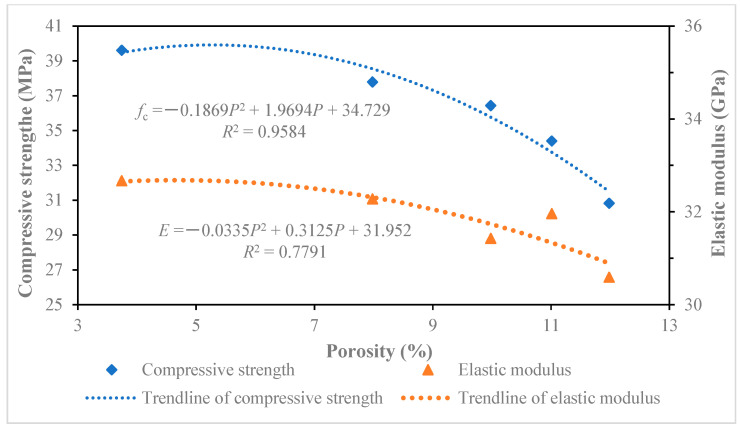
Effect of pores 50~200 nm on compressive strength and elastic modulus of recycled concrete.

**Figure 13:**
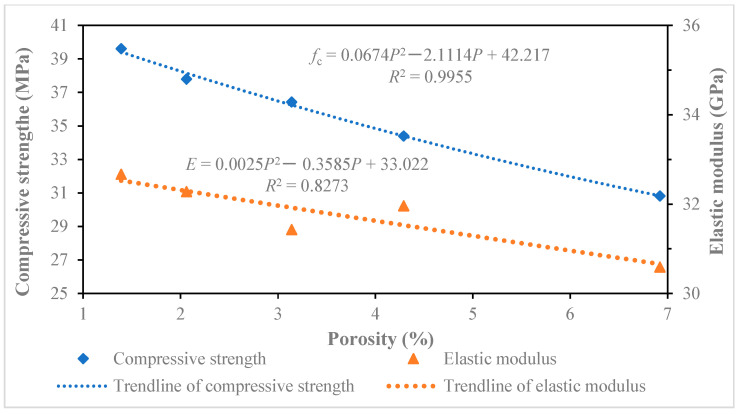
Effect of pores above 200 nm on compressive strength and elastic modulus of recycled concrete.

By further analyzing the data in Table 5, the relationship curves of different pore sizes between the compressive strength and elastic modulus of recycled concrete were drawn, as shown in Figure 10, Figure 11, Figure 12 and Figure 13. The results of the relationship curves showed that the relationship between compressive strength and elastic modulus was a quadratic polynomial, the fitting degree of the data was good and the regression coefficient was high. The compressive strength and elastic modulus of recycled concrete increased with the increase in porosity of below 20 nm and 20~50 nm pores. The compressive strength and elastic modulus of recycled concrete decreased with the increase of porosity in 50–200 nm and above 200 nm pores.

## 6. Quantitative Characterization of Compressive Strength or Elastic Modulus of Recycled Concrete Based on Pore Grading

### 6.1. Establishment of Quantitative Model

Above, the correlation and influence trend of the porosity of different pore sizes on compressive strength and elastic modulus of recycled concrete were analyzed. It was also necessary to establish a quantitative relationship model of compressive strength or elastic modulus of recycled concrete based on pore grading. For this reason, the least square method was used to analyze the data in Table 5. The quantitative relationship of compressive strength or elastic modulus of recycled concrete based on hole grading is shown in Equation (5).

(5) $$\left\{\begin{array}{c} \begin{array}{ll} f_{c}= & 34.90+3.7649P_{<20}-0.2843P_{<20}^{2}-0.6093P_{20-50}-0.1476P_{20-50}^{2}\\ & +1.2021P_{50-200}-0.0604P_{50-200}^{2}-0.7048P_{\geq 200}-0.0610P_{\geq 200}^{2} \end{array}\\ \begin{array}{ll} E= & 19.06+4.5516P_{<20}-0.2866P_{<20}^{2}-0.4509P_{20-50}-0.1518P_{20-50}^{2}\\ & +2.2067P_{50-200}-0.0731P_{50-200}^{2}-0.2036P_{\geq 200}-0.0343P_{\geq 200}^{2} \end{array} \end{array}\right.$$

### 6.2. Error Analysis Between Test Results and Predicted Results

The results of the quantitative relationship model predicting compressive strength and elastic modulus were compared with the experimental results, as shown in Table 9. From the comparison results, it can be seen that the predicted results are in good agreement with the test results, the test results are close to the predicted results, and the maximum error is 1.66%. Therefore, the established quantitative relationship model is reasonable.

### 6.3. Comparative Analysis Between Quantitative Characterization Model and Existing Models

The experimental data were replaced by the existing strong-pore model, and the parameters in the model were re-calibrated by the regression analysis method. Calibration results are shown in Table 8.

According to Table 10, the quantization model of compressive strength and elastic modulus of recycled concrete based on pore grading has higher correlation coefficients than Hensselmann [21], Schille [22], Ryshkewitch [23] and Powers [6]. This was partly due to the fact that the addition of recycled coarse aggregate led to a more complex concrete micro-pore size, which reduced the accuracy of the strength-pore model. Considering the different effects of different pore sizes on the compressive strength and elastic modulus of recycled concrete, the results of compressive strength and elastic modulus of recycled concrete can be predicted more accurately.

It can be seen from the above analysis that the effect of large pore sizes on the compressive strength and elastic modulus of recycled concrete is more unfavorable, and reducing the proportion of large pore sizes and total porosity of recycled concrete is conducive to improving its compressive strength and elastic modulus. Therefore, measures should be taken to reduce the proportion of large pore size in the production of recycled concrete, such as extending the shaking time and strengthening the treatment of recycled coarse aggregate.

## 7. Conclusions

From the above experimental research and analysis results of recycled concrete, the following conclusions can be drawn.

(1) With the increase in the replacement rate of recycled coarse aggregate, the compressive strength and elastic modulus of recycled concrete decreased, and the greater the replacement rate, the more obvious the decrease.
(2) With the increase in the replacement rate of recycled coarse aggregate, the peak pore size in the pore size distribution curve of recycled concrete increased continuously, and the proportion of pore size above 50~200 nm and 200 nm also increased continuously.
(3) The pore size below 20 nm and 20~50 nm had a positive correlation with the compressive strength and elastic modulus of recycled concrete, while the pore size above 50~200 nm and 200 nm had a negative correlation with the compressive strength and elastic modulus of recycled concrete. The test results of recycled concrete verify that the quantitative characterization model could better characterize the compressive strength and elastic modulus of recycled concrete.

## Figures and Tables

**Table 1:** Basic physical parameters of NCA and RCA.

Coarse Aggregate	Bulk Density (kg/m^3^)	Apparent Density (kg/m^3^)	Crush Value (%)	Water Absorption (%)
NCA	1257	2673	6.4	0.85
RCA	1123	2492	13.2	3.75

**Table 2:** Basic physical properties of the cement.

Specific Surface Area (m^2^/kg)	Firing Loss (%)	Fineness (μm)	Standard Consistency (%)	Setting Time (h:min)		Compressive Strength (MPa)		
				Initial Set	Final Set	3d	7d	28d
333.3	2.14	32	27.9	2:35	3:56	25.9	34.1	51.4

**Table 3:** Main chemical composition of the cement (%).

SiO_2_	Al_2_O_3_	Fe_2_O_3_	CaO	MgO	SO_3_	K_2_O	Na_2_O	TiO_2_	P_2_O_5_
24.61	7.64	2.8	56.27	2.39	2.17	1.45	0.42	0.3	0.05

**Table 4:** The mix of the recycled concrete (kg).

Specimen	Replacement Rate	Water	Cement	RCA	NCA	Sand	Water Reducing Agent	Additional Water	Slump/mm
RC10	10%	140	348	1202	134	580	0.976	0	63
RC20	20%	140	348	1068	267	580	1.114	+4.0	61
RC30	30%	140	348	935	401	580	1.198	+7.0	60
RC40	40%	140	348	801	534	580	1.375	+11.0	59
RC60	60%	140	348	534	803	580	1.537	+19.0	56

**Table 5:** Parameters of CPMG sequence.

System Parameter	Parameter Value	System Parameter	Parameter Value
SF	21 MHz	O1	275,415.9 Hz
RFD	0.100 ms	P1	17.00 μs
TW	2000 ms	P2	32.48 μs
TD	44,994	NS	2
SW	100 kHz	TE	0.300 ms
RG1	10 db	NE	1500
DRG1	3	Pore type	Cylindrical or tubular
PRG	1	Permeation model	Coates model

**Table 6:** The average values and standard deviations of the compressive strength and elastic modulus.

Recycled Concrete	Compressive Strength		Elastic Modulus	
	Average Value (MPa)	Standard Deviation	Average Value (GPa)	Standard Deviation
RC10	39.61	1.55	32.67	1.58
RC20	37.79	0.64	32.28	0.72
RC30	36.43	0.54	31.43	0.52
RC40	34.4	0.78	31.96	0.65
RC60	30.82	1.15	30.59	1.21

**Table 7:** The porosity of recycled concrete with different pore sizes.

Recycled Concrete	Compressive Strength (MPa)	Elastic Modulus (GPa)	Porosity of Different Pore Sizes (%)			
			<20 nm	20~50 nm	50~200 nm	>200 nm
RC10	39.61	32.67	5.19	6.29	3.74	1.39
RC20	37.79	32.28	2.13	5.62	7.98	2.06
RC30	36.43	31.43	1.17	4.81	9.98	3.14
RC40	34.40	31.96	0.78	4.14	11.01	4.29
RC60	30.82	30.59	0.40	3.26	11.98	6.92

**Table 8:** Correlation between different pore sizes and compressive strength or elastic modulus of recycled concrete.

Item	Different Pore Size			
	<20 nm	20~50 nm	50~200 nm	>200 nm
Compressive strength	0.827	0.987 **	−0.887 *	−0.995 **
Elastic modulus	0.782	0.893 *	−0.827	−0.910 *

Note: ** The correlation was significant at level 0.01 (two-tailed). * The correlation was significant at level 0.05 (two-tailed).

**Table 9:** Comparison between test results and predicated results.

Compressive Strength (MPa)			Elastic Modulus (GPa)		
Test Results	Predicted Results	Error	Test Results	Predicted Results	Error
39.61	39.65	0.10%	32.67	32.71	0.12%
37.79	37.89	0.26%	32.28	32.09	−0.59%
36.43	36.25	−0.49%	31.43	31.78	1.10%
34.4	34.61	0.61%	31.96	31.43	−1.66%
30.82	30.71	−0.36%	30.59	30.40	−0.62%

**Table 10:** Comparison of models for strength-pore structure relationship.

Proposer	Equation After Calibration	*R* ^2^
Model in this paper	$\left\{\begin{array}{l} \begin{array}{ll} f_{c}= & 34.90+3.7649P_{<20}-0.2843P_{<20}^{2}-0.6093P_{20-50}-0.1476P_{20-50}^{2}\\ & +1.2021P_{50-200}-0.0604P_{50-200}^{2}-0.7048P_{\geq 200}-0.0610P_{\geq 200}^{2} \end{array}\\ \begin{array}{ll} E= & 19.06+4.5516P_{<20}-0.2866P_{<20}^{2}-0.4509P_{20-50}-0.1518P_{20-50}^{2}\\ & +2.2067P_{50-200}-0.0731P_{50-200}^{2}-0.2036P_{\geq 200}-0.0343P_{\geq 200}^{2} \end{array} \end{array}\right.$	0.9954 0.9962
Hensselmann	$f_{c}=63.743-1.452P$	0.6944
Schille	$f_{c}=118.67-28.08\ln(P)$	0.6872
Ryshkewitch	$f_{c}=79.233\exp(-0.041P)$	0.6896
Powers	$f_{c}=378.66P^{-0.8}$	0.6782

## Data Availability

The original contributions presented in the study are included in the article, further inquiries can be directed to the corresponding authors.
